# The Impact of a Web-Based Mindfulness, Nutrition, and Physical Activity Platform on the Health Status of First-Year University Students: Protocol for a Randomized Controlled Trial

**DOI:** 10.2196/24534

**Published:** 2021-03-10

**Authors:** Claire F Trottier, Jessica R L Lieffers, Steven T Johnson, João F Mota, Roshni K Gill, Carla M Prado

**Affiliations:** 1 Human Nutrition Research Unit Department of Agricultural, Food and Nutritional Science University of Alberta Edmonton, AB Canada; 2 College of Pharmacy and Nutrition University of Saskatchewan Saskatoon, SK Canada; 3 Faculty of Health Disciplines Athabasca University Athabasca, AB Canada; 4 Clinical and Sports Nutrition Research Laboratory (LABINCE) Faculty of Nutrition Federal University of Goiás Goiânia Brazil

**Keywords:** internet-based intervention, wellness programs, dietary intake, physical activity, mindfulness, quality of life, randomized controlled trial

## Abstract

**Background:**

First-year university students are at an increased risk for developing mental health issues and a poor nutritional status. Self-care plays an essential role in optimizing mental health and can prevent or manage stress, anxiety, and depression. Web-based self-monitoring of diet and physical activity can lead to similar or improved health outcomes compared with conventional methods. Such tools are also popular among university students.

**Objective:**

The primary aim of this 12-week randomized controlled trial is to assess the impact of a web-based wellness platform on perceived stress among first-year university students. The secondary aim is to assess the effects of the platform on diet quality. The exploratory objectives are to explore the effects of the platform on body composition, health-related quality of life, mindfulness, mental well-being, and physical activity.

**Methods:**

A total of 97 first-year undergraduate students were randomized to either the intervention (n=48) or control (n=49) group. The intervention consisted of access to a web-based platform called My Viva Plan (MVP), which aims to support healthy living by focusing on the topics of mindfulness, nutrition, and physical activity. The platform is fully automated and guided by the principles of cognitive behavioral theory. Participants in the intervention group were instructed to use the MVP as frequently as possible over 12 weeks. The control group did not receive access to MVP. Perceived stress was assessed using the Stress Indicators Questionnaire at baseline, week 6, and week 12. Three-day food records were used to analyze the dietary intake at baseline and week 12. Health-related quality of life, mindfulness, mental well-being, and physical activity questionnaires were completed at baseline, week 6, and week 12. Body composition was assessed at baseline and week 12. Study assessments were completed in person at baseline and week 12 and electronically at week 6.

**Results:**

Study recruitment started in August 2018, with batch enrollment for students registered in the fall (September 2018 to December 2018) and winter (January 2019 to April 2019) academic terms at the University of Alberta, Edmonton, Alberta.

**Conclusions:**

This study is the first to explore the impact of a web-based platform designed to promote health and wellness on perceived stress and diet quality among first-year university students.

**Trial Registration:**

ClinicalTrials.gov NCT03579264; https://clinicaltrials.gov/ct2/show/NCT03579264.

**International Registered Report Identifier (IRRID):**

DERR1-10.2196/24534

## Introduction

### Background

University students are at risk of developing mental [1] and physical [2-4] health problems. The number of students reporting symptoms of anxiety [5], depression [5], and suicidal thoughts [1,6] continues to rise, and this is a greater concern among first-year undergraduate students [7]. During this important yet vulnerable period of their lives, students not only transition from adolescence to adulthood but also start to face challenging situations and the new responsibilities of career development and a self-sufficient life [8]. This specific period and situation is related to neurobiological, behavioral, and environmental changes that impose significant psychological stress [9,10] and an increase in the risk of psychiatric disorders [11].

In addition to mental health, during the first year of a university program, students’ dietary habits and physical activity are also negatively impacted [2,3,12,13]. Students increase their consumption of unhealthy, energy-dense foods and reduce their intake of healthy, nutrient-dense foods in their first year of university, resulting in an overall decrease in diet quality [13]. These changes may result in unfavorable alterations of their nutritional status, such as an increase in body weight and fat mass [3,13,14]. In a meta-analysis of 5549 university students, the mean weight change during the first year was 1.36 kg (95% CI 1.15-1.57 kg) [15]. Hoffman et al [3] observed an increase of 0.7% of fat mass among first-year university students, which further illustrates the physical changes that are observed in this population.

Increased psychological stress and poor nutritional status can hinder students’ quality of life [16], hinder academic performance [17], and increase morbidity [18] and mortality [19]. Apart from the direct impact on students, mental health problems in the general population and workforce impose a significant financial burden on the health care system [20,21]. Universities may be the last setting in which mental health programs can be promoted to a large portion of young adults before they enter the diversified workforce [22]. Therefore, the development of programs focused on the mental and physical health status of university students is of special interest.

University students rely heavily on technology to perform tasks of daily living, and health care is no exception [23]. The use of electronic tools to monitor health status is increasing in the general population. Of the expected 80-100 billion smart devices in the world by 2020, approximately 26 billion will be used to monitor aspects of personal health and well-being [23]. Therefore, individualized technological tools encompassing aspects of practicality, availability, cost, and time efficiency are promising health promotion approaches for this generation (ie, generation Z).

Research has shown that electronic monitoring of diet or diet and physical activity can lead to similar or better health outcomes compared with conventional methods such as paper-based food records and weight loss resulting from counseling with practitioners [24-27]. Although digital health apps for nutrition, physical activity, and mental health are extremely popular among young adults [28], many of these tools have not been designed by health care professionals [29,30] and lack a sound evidence base [31]. Moreover, previous research has shown that poor integration between nutrition and physical activity apps or trackers is a concern among users [32].

Considering the limitations of many available eHealth tools, My Viva Plan (MVP; Multimedia Appendix 1) [33] addresses some common shortfalls as it was developed by a registered dietitian (Loreen Wales, Revive Wellness Inc) in collaboration with other health care professionals (registered psychologists, certified personal trainers, and kinesiologists). It focuses on multiple pillars of health: mindfulness, nutrition, and physical activity. MVP is a publicly accessible web-based program available via paid subscription. The platform can be used as a stand-alone intervention or in combination with coaching from professionals. MVP has 3 variations of the platform to target different populations: 1 variation targeted at the general population and 2 variations targeted at students attending local postsecondary institutions. The variation used and described in this study is the variation that is targeted at students attending the University of Alberta (Edmonton, Alberta).

### Objectives and Hypothesis

The main objective of this 12-week randomized controlled trial is to assess the impact of MVP on perceived stress among first-year university students. The secondary objective is to assess the impact on diet quality, and exploratory objectives will assess the impact on body composition, health-related quality of life, mindfulness, mental well-being, and physical activity. It was hypothesized that using an integrated web-based wellness platform that encompasses the 3 pillars of preventative self-care (mindfulness, nutrition, and physical activity) would help guide health behavior, resulting in improved perceived stress and diet quality in first-year university students.

## Methods

### Study Design and Ethical Considerations

This 2-armed, parallel, randomized controlled trial was conducted at the Human Nutrition Research Unit (HNRU), University of Alberta, and used an embedded mixed methods design [34,35]. The research protocol is approved by the University of Alberta Ethics Board (Pro00079680) and complies with the standards set out in the Canadian Tri-Council Policy statement on the use of human participants in research. Before the commencement of the study, all participants were informed in person of the procedures and potential risks involved in the investigation and were asked to provide written informed consent. All participants, regardless of group assignment, received CAD $50 (US $39) cash and one year of free membership to MVP after completing all study assessments.

### Research Participants

First-year undergraduate students at the University of Alberta were recruited using a variety of techniques (in-class invitations, in person through flyers distributed at special events and locations, email, social media, and advertisements placed on notice boards on campus) during the fall (September 2018 to December 2018) and winter (January 2019 to April 2019) terms. Various campus faculties were targeted for recruitment to ensure a diverse sample. Eligible participants were males and females aged 17-30 years who were able to complete all study assessments in their first year of undergraduate studies. Reasons for exclusion included any self-reported eating disorder; self-reported untreated depression, anxiety, or mood disorder; pregnancy or lactation; not having a device to access the internet (ie, computer, smartphone, tablet); not able to communicate in English; or having an electronic implantable device (because of body composition assessment). Participants were assumed to have strong computer and internet literacy, given the typical age and education level of the study population; however, literacy levels of the participants were not assessed.

### Screening, Randomization, and Treatment Allocation

Potential participants contacted the research team and underwent prescreening using the same method in which they contacted the research team (ie, via email or over phone). The research team discussed the responsibilities of the participants (ie, time commitment of each study visit and a commitment of 12 weeks of participation) and addressed any questions before scheduling the baseline visit. The screening criteria were later confirmed and documented in person at the baseline visit. A member of the research team reviewed the study consent form in detail with each participant during their baseline visit, and participants received a copy of their signed study consent form for their records. All eligible and consenting participants were randomly assigned by block randomization to the intervention (platform use) or control (no intervention) group in a 1:1 allocation ratio. Blocks were stratified by sex to balance sex distribution and block sizes randomly alternated between blocks of 2 and 4 to minimize the risk of predictable group allocation [36].

The allocation sequence was generated by an independent biostatistician and uploaded onto Research Electronic Data Capture (REDCap) [37,38], where it was concealed from the study team. The study team enrolled and assigned the participants to the groups using REDCap. The participants and researchers were not blinded to the group assignment because of the nature of the study. The staff needed to know the participants’ group assignment to provide access to the platform, monitor use, and schedule interviews.

### Experimental Protocol

**Figure 1:**
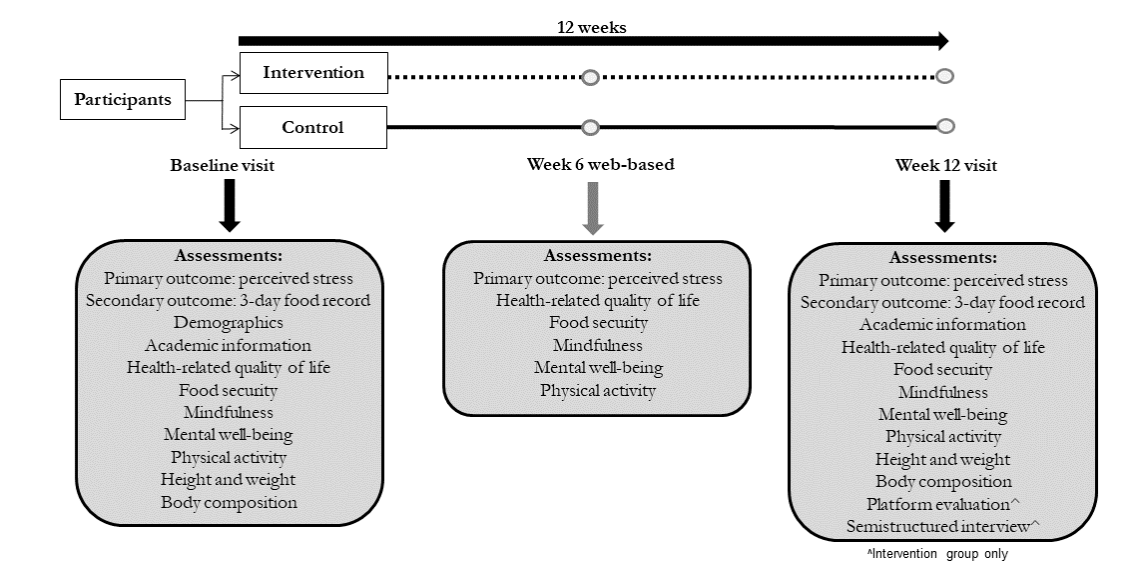
Outline of the experimental protocol.

Participants allocated to the intervention group were instructed to use MVP as frequently as possible for the duration of the 12-week period. Participants allocated to the control group were asked to maintain their usual lifestyle throughout the study and did not receive access to MVP during the 12 weeks of study participation. In-person study visits occurred at baseline and week 12 at the HNRU (Figure 1), and the following assessments were collected: academic information, anthropometry, body composition, demographics (at baseline only), dietary intake, food security, health-related quality of life, mindfulness, mental well-being, perceived stress, and physical activity (Multimedia Appendix 2). At week 6, questionnaires were electronically administered (Figure 1) and collected via web-based surveys through REDCap [37,38] to assess food security, health-related quality of life, mindfulness, mental well-being, perceived stress, and physical activity (Multimedia Appendix 2). Participants assigned to the intervention group were asked to evaluate MVP by questionnaire at their week 12 visit and participate in a one-on-one semistructured interview to gather qualitative information on their experiences with and perceptions of MVP. Details of the qualitative analysis are published in a separate publication.

### Intervention

The principles of cognitive behavioral theory (CBT) [39], specifically the social cognitive theory of self-regulation [40], were used to guide the development of the MVP platform. The goal is to build resilience and self-efficacy through the development of strong self-care habits. The platform included features such as goal setting, diet plans, exercise tutorials, and self-monitoring of health behaviors (ie, stress levels). The variation of the platform used in our study was fully automated, with no human interaction. The University of Alberta logo is displayed on the log-in page of MVP and on any exercise video made in collaboration with the University of Alberta’s Campus and Community Recreation facilities; however, institutional logos were not present anywhere else within the platform.

All participants in the intervention group received free access to the same version of the MVP, and membership access was delivered by email within 5 days of completing baseline assessments. While using the platform, users were prompted to create a profile and fill in the basic medical history questions, including food allergies, medical conditions, and current medications. A video tutorial provided guidance on the platform and recommended that new users start with the mindfulness component. The tutorial explained that each component of the platform can be used independently or in combination and that users could set up a shortcut on their mobile device to directly access the MVP platform. No additional MVP training was provided by the researchers.

The 3 core components of MVP are described as follows:

1. My Mind encourages goal setting, daily reflections, and quarterly stress assessments. One of the first steps in setting up an MVP account is the goal-setting process. The platform allows users to set a maximum of 3 short-term and 3 long-term goals. Participants set their own goals, which could be modified at any time. The platform also advises users to keep their goals specific, measurable, achievable, realistic, and time-oriented. Examples of goals include “I will stretch every night before bed” (short term) and “I will train for a marathon this year” (long term). Daily reflections on stress levels, eating habits, physical activity, and steps taken toward achieving goals are encouraged. On the basis of the responses from the daily reflections, the user’s progress is viewable in a graphical format (such as a bar graph to indicate the level of stress on each day of the week and a checkmark on days of the week that steps were taken to achieve goals).
2. My Nutrition contains meal plans, recipes, grocery shopping list, cooking demonstrations, Vivapedia (an encyclopedia on the nutritional benefits of numerous food items and nutrients), and a guide with suggested menu items from restaurants and fast-food chains in Edmonton and on the University of Alberta’s campus. Meal plans allow users to create their own plans for meals each day or to have a plan built for them. When a user builds their own meal plan, the number of suggested portions of protein, grains, fruits, vegetables, dairy, and fat are generated by the platform to fit the recommendations of the Eating Well with Canada’s Food Guide [41], and the user can choose foods from each food group, either from a drop-down list or by manually entering the food item, to reach these targets. Menu plans built by the platform for the user are based on the user’s preferences and meet the recommendations of the 2007 Eating Well with Canada’s Food Guide [41]. Platform-derived meal plans can be customized for allergies, intolerances, and other food preferences (ie, vegetarian, gluten-free, nut-free, dairy-free) and provide brief instructions on how to prepare each meal. A serving size guide is available within the platform and as a printable handout. The recommended proportion of foods from each food category is represented as a dinner plate model for users to refer.
3. My Fitness provides a physical activity scheduler and video tutorials for workouts and yoga. The workout tutorials have accommodations for self-described fitness level, equipment availability, and desired duration, whereas the yoga tutorials incorporate guided meditations and can be adjusted by self-described fitness level and desired duration. Video workouts included resistance training, aerobic exercises, and stretching. In addition, users can log the duration and intensity of their workouts completed outside the platform.

To help foster learning in a wide range of individuals, the platform provides educational resources in multiple formats, including video content and printable handouts. MVP employs strategies commonly used in clinical practice, such as CBT, into daily reflections of health behaviors. Outside of the platform, coaching tips that provide messages of healthy living (ie, being present, seeking accountability, healthy snacking, keeping active) are emailed on a weekly basis and presented in the same sequence for all users. Users also receive student-specific messages in key times throughout the academic year (ie, managing the first week of a new academic term, preparing for midterm exams, preparing for final exams). The content of these email messages is not tailored based on the user’s activity within the platform.

### Assessment of Study Outcomes

#### Primary Outcome

Our primary outcome is the difference in change over time in perceived stress (baseline to week 12) between the groups. Perceived stress was measured using the self-reported Stress Indicator Questionnaire [42]. Total scores range from 73 to 365 points, with higher total scores indicating higher levels of perceived stress.

#### Secondary Outcome

Our secondary outcome is the difference in the change of diet quality between the groups. Dietary intake was assessed using paper-based 3-day food records that were analyzed using Food Processor Nutrition Analysis Software, version 11.0.3 (ESHA Research). Participants were asked to enter information about all the food, beverages, and supplements they consumed over 3 days, with 2 of the days on a weekday and 1 of the days on a weekend day. Instructions on how to complete the food records were included at the beginning of the paper food record booklet. In addition, instructions on how to complete food records were provided in person with participants during their baseline visit.

Diet quality was estimated using the nutrient rich food (NRF) index version 9.3 [43,44]. This index calculates a nutrient density score based on the amount of nutrients per 100 kcal serving of an individual food item: saturated fat, sodium, total sugar, protein, fiber, iron, magnesium, calcium, potassium, and vitamins A, C, and D. The percent daily value was calculated for all the nutrients. The sum of the percent daily values of nutrients to limit (saturated fat, sodium, and total sugar) was subtracted from the sum of nutrients to encourage (protein, fiber, iron, magnesium, calcium, potassium, and vitamins A, C, and D) to obtain the NRF score for that food item.

Vitamin D was chosen to replace vitamin E because it has been identified as an important nutrient for encouraging [45,46]. For protein, the lower limit of 10% of calories from protein in a 2000-kcal diet was used based on the acceptable macronutrient distribution ranges [47]. The percent daily values used in this study were 20 g saturated fat, 2400 mg sodium, 100 g total sugar, 50 g protein, 25 g fiber, 14 mg iron, 420 mg magnesium, 1100 mg calcium, 4700 mg potassium, 1000 retinol equivalent vitamin A, 60 mg vitamin C, and 600 international unit vitamin D.

To limit the effect of fortified foods, percent daily values were capped at 100%. A low or negative NRF score indicates a low-nutrient–dense food, and a high or positive NRF score indicates greater nutrient density. The total score was calculated by averaging all individual food scores within a 3-day food record.

#### Exploratory Outcomes

The following exploratory outcomes were assessed and will be compared within groups (over time) and between groups at the end of the 12-week intervention period:

- Body composition was assessed by a hand-to-foot multifrequency bioelectrical impedance analysis using the QuadScan 4000 (BodyStat). Participants were instructed to fast for 5 hours before testing (drinking water only) and to avoid intense physical activity for 12 hours. Immediately before testing, participants were instructed to remove their shoes, socks, and all metal accessories before lying in a supine position. Fat mass, fat-free mass, and total body water were estimated based on device-specific equations.
- Health-related quality of life was assessed using the self-administered 12-Item Short-Form Health Survey version 2 [48]. Physical and mental component summary scores on a scale of 0 to 100 were generated and interpreted against data from the general population of the United States. Higher scores indicate a greater quality of life [49], and a difference in summary scores between 3 and 5 points is considered clinically significant [50].
- Mindfulness was assessed using the self-administered Five Facet Mindfulness Questionnaire (FFMQ) [51]. The FFMQ contains 39 statements about 5 facets of mindfulness (observation, description, awareness actions, nonjudgment, and nonreactivity) that are rated and scored on a 5-point Likert-type scale from never true to always true. Construct validity of the FFMQ has been demonstrated [52].
- Mental well-being was measured using the self-administered Warwick-Edinburgh Mental Wellbeing Scale [53]. The 14 questions on aspects of mental well-being were assessed using a 5-point Likert scale that was used to score each question and summed for a total score, with a higher score indicating higher mental well-being [53].
- Physical activity was assessed using a validated, self-administered Godin-Shephard Leisure-Time Physical Activity Questionnaire [54]. The number of times in a typical week that strenuous, moderate, and mild or light activity longer than 15 minutes during free time was reported. A score was calculated based on the number of times per week multiplied by an intensity factor (9 for strenuous, 5 for moderate, and 3 for mild or light). On the basis of the total score, the amount of activity was classified as active, moderately active, or sedentary [55].

#### Other Assessments

Demographic information collected included sex, age, ethnicity, and current type of residence. Self-reported academic information collected included the degree type and the number of enrolled courses. Academic performance was evaluated based on students’ self-reported grades. Trained staff measured the participant’s anthropometrics and instructed participants to wear light clothing and remove their footwear. The body weight was measured using a calibrated digital scale (Health o meter Professional Remote Display, Sunbeam Products Inc) to the nearest 0.1 kg. Height was measured using a 235 Heightronic Digital Stadiometer (Quick Medical) to the nearest 0.1 cm. BMI was calculated and evaluated using previously defined categories [56]. Food security was assessed using a 6-question survey, as described by Entz et al [57].

Participants in the intervention group were asked to evaluate MVP at the week 12 visit using the Mobile App Rating Scale questionnaire [58]. Subscales for the following sections were calculated: engagement, functionality, esthetics, and information quality. To account for possible ratings of *not applicable*, the mean is calculated for each subscale and summed to generate a mean total score, where a higher total mean score represents a positive evaluation.

### Intervention Preference and Website Usage Data

System-obtained data were collected directly from the MVP for participants allocated to the intervention group. These data included information on the total number of log-ins, duration of activity, and usage of specific components of the app (ie, frequency of daily reflections, frequency of planned physical activities). A member of the study team reviewed participants’ platform usage every 3 weeks to assess the log-in activity. Individuals who had been identified as not logging on to the platform in that 3-week period were contacted by a member of the research team via email or over phone to ask if their ability to access the platform had been impeded and to act as a reminder of their agreement to use the platform as frequently as possible.

### Data Management

Study data were collected and managed using REDCap electronic data capture tools hosted at the University of Alberta [37,38]. REDCap is a secure, web-based software platform designed to support data capture for research studies, providing (1) an intuitive interface for validated data capture, (2) audit trails for tracking data manipulation and export procedures, (3) automated export procedures for seamless data downloads to common statistical packages, and (4) procedures for data integration and interoperability with external sources. Data collected using paper files at baseline and week 12 were transferred into REDCap and reviewed by an independent researcher within the team to identify data entry errors. At week 6, the study questionnaires were delivered electronically through REDCap and collected directly from the participant. Platform-obtained data were managed using Microsoft Excel (Microsoft Corporation).

### Statistical Analysis and Power and Sample Size Rationale

#### Statistical Analysis

Our analysis of the primary outcome will be based on an intention-to-treat analysis using inverse probability weighting. Our analysis of the secondary and exploratory outcomes will be based on per-protocol and intention-to-treat analyses using inverse probability weighting. The effects of intervention over time will also be investigated using a generalized estimating equation. SPSS 25 (IBM Corporation) statistical software will be used for the data analysis. Characteristic variables will be compared between the 2 treatment groups and between those that did and did not complete the study. Differential variables will be used as covariates in the statistical analyses. *P*<.05 will be considered statistically significant.

#### Power and Sample Size

The sample size was calculated using G-Power software (version 3.1.9.2, Heinrich-Heine-Universität Düsseldorf) [59] and based on the perceived stress scale scores of distressed adults after an internet-based stress management intervention [60]. With effect sizes of 0.74, a level of significance of 5%, and statistical power of 95% (power=1-β=0.95), the number of participants required is 33. With an anticipated attrition rate of 20%, we aimed to recruit 100 participants and have 80 participants complete the intervention.

## Results

**Figure 2:**
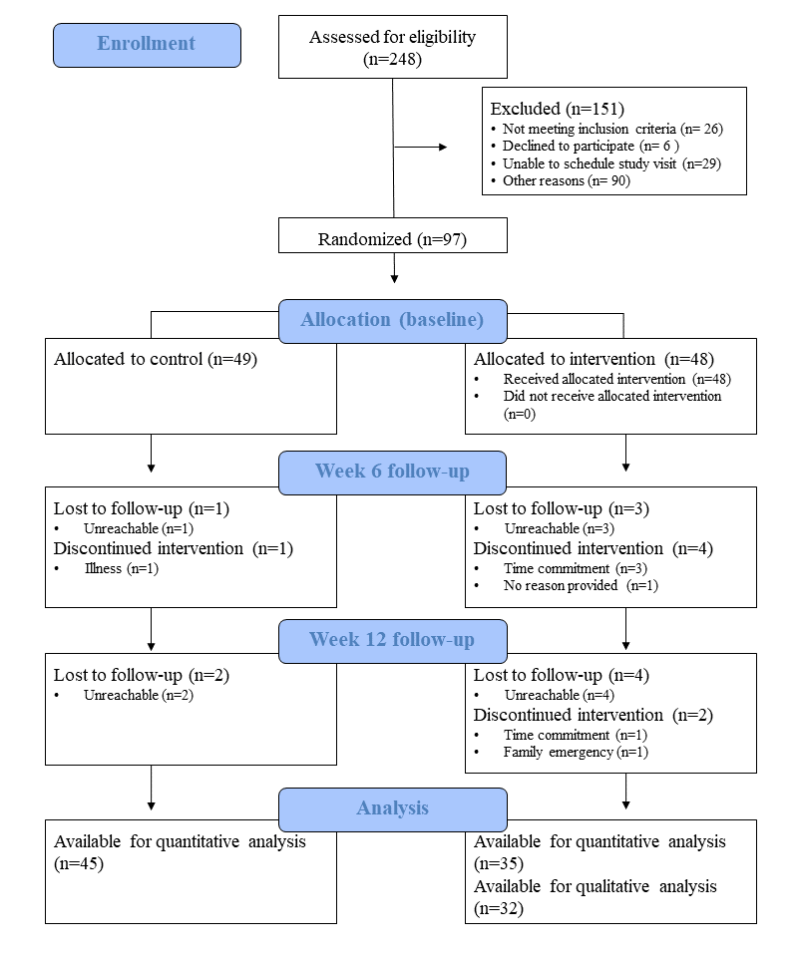
Flow diagram.

Study recruitment began in August 2018. Batch recruitment was used for enrollment in the fall (September 2018 to December 2018) and winter (January 2019 to April 2019) terms, with 37 and 43 participants completing the study in the fall and winter terms, respectively. A total of 80 participants completed the 12-week trial (n=35 in the intervention group; n=45 in the control group; Figure 2).

## Discussion

### Strengths

This study used a mixed methods design to capture both quantitative and qualitative findings from first-year university students’ experiences using MVP and is comprehensive in the variety of data captured. The MVP platform itself has a different approach than other web-based tools as it focuses on multiple pillars of health (mindfulness, nutrition, and physical activity), provides a comprehensive list of resources to its users, is developed by certified health professionals, and is guided by principles of CBT. In addition, the issues commonly experienced by university students were strongly considered in the development of MVP. For example, MVP delivered messages about stress management during key times in the academic term and guides to healthy eating on campus, further supporting healthy food choices.

### Limitations

Potential limitations of the study are related to the inclusion of only first-year university students and the monitoring of platform usage, which may have limited external validity. Attrition was higher in the group that received the intervention than in the control group, although platform usage data have not yet been analyzed. This suggests that systematic differences between groups should be examined in outcome analyses. Finally, all limitations will be examined and taken into account in the interpretation of the results.

### Conclusions

Preventative self-care is important for first-year university students who may not have established this skill and are simultaneously experiencing new stress and pressures of postsecondary education. Targets were obtained for the sample size. This study will determine whether the web-based platform has a direct impact on health and wellness among first-year university students and could become an important tool for virtual, preventative self-care delivery.

## Appendix

Multimedia Appendix 1 Screenshots of My Viva Plan.

Multimedia Appendix 2 Study timeline.

## Abbreviations

CBT: cognitive behavioral theory
FFMQ: Five Facet Mindfulness Questionnaire
HNRU: Human Nutrition Research Unit
MVP: My Viva Plan
NRF: nutrient rich food
REDCap: Research Electronic Data Capture
