# Supplementary material for: The Impact of a Web-Based Mindfulness, Nutrition, and Physical Activity Platform on the Health Status of First-Year University Students: Protocol for a Randomized Controlled Trial
Source: JMIR Res Protoc. 2021 Mar 10;10(3):e24534. doi: 10.2196/24534 (PMC7991982; doi:10.2196/24534)
Supplement: Multimedia Appendix 1 [file resprot_v10i3e24534_app1.pdf]

WHOLE BODY HEALTH

My Viva Inc. is a community for a better you. To us, Whole Body Health means taking care of how you engage your mind, fuel your body, and move your body.

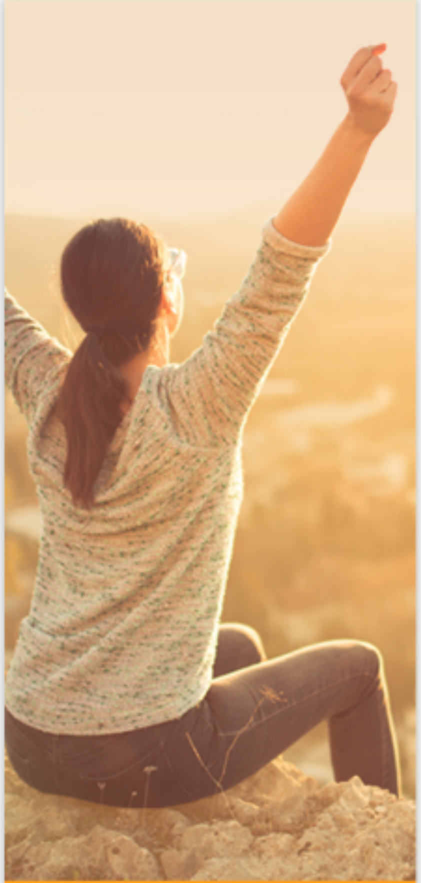

Mindfulness

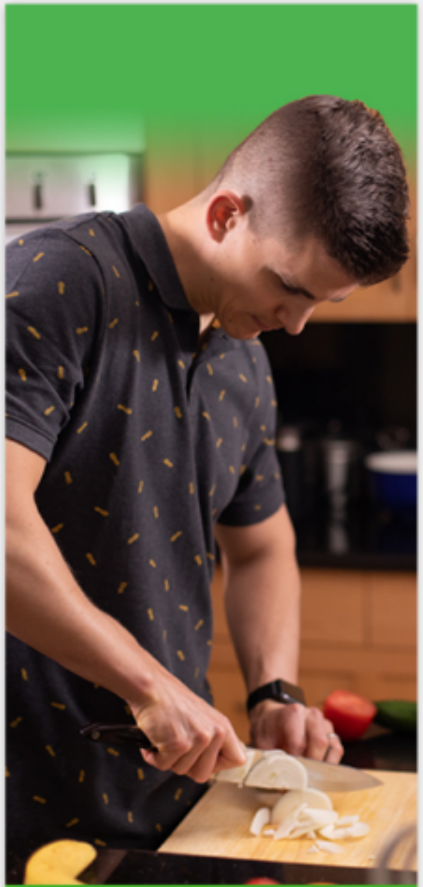

Nutrition

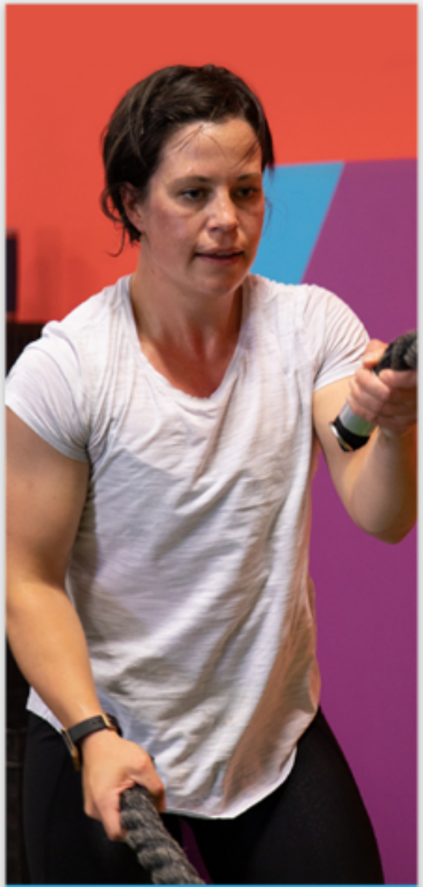

Fitness

RECHARGE
your wellness, for a better you.

UALBERTA MY VIVA PLAN

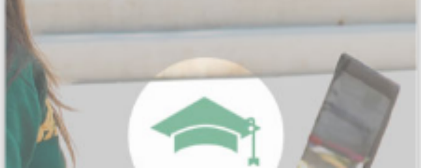

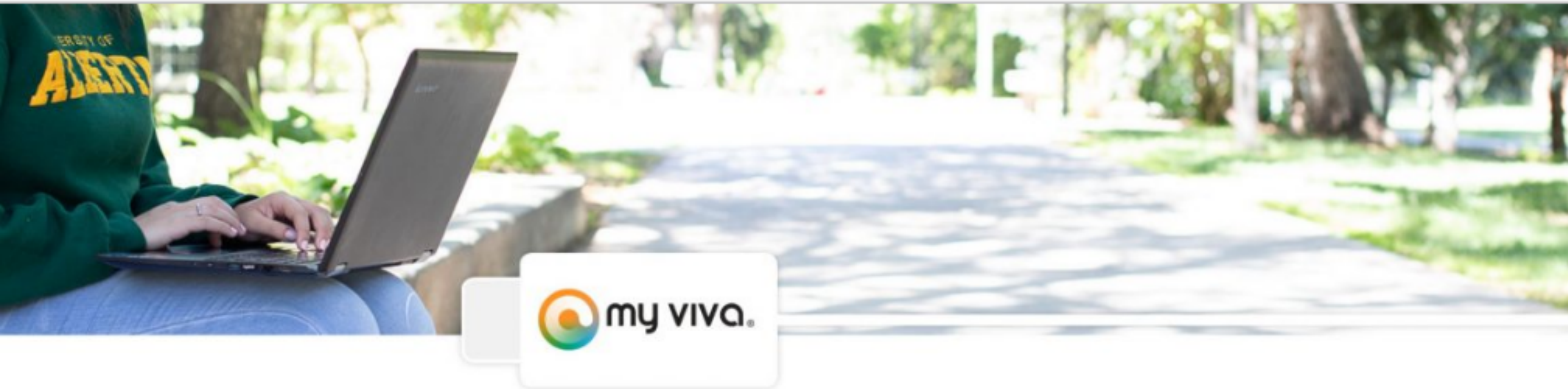

STAY HEALTHY ON CAMPUS WITH MY VIVA PLAN!

Being a student at a university or post-secondary institution adds a lot of stress to your life. From attending classes to studying to working a part-time job, there are many things that you need to focus on while on campus. This busy schedule can make it hard to stay healthy mentally, physically, and emotionally. It can also be difficult to eat a healthy diet when fast food and instant meals are easier than cooking for yourself.

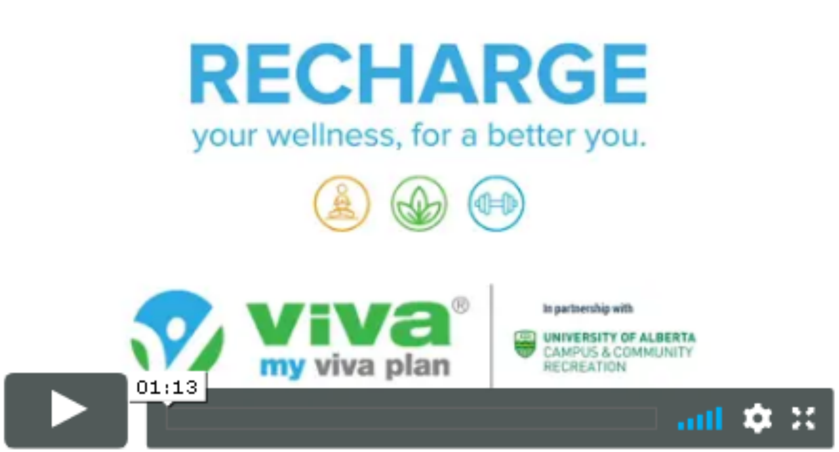

Our health and wellness plans focus on nutrition, fitness, and mental wellness, helping to create whole body health.

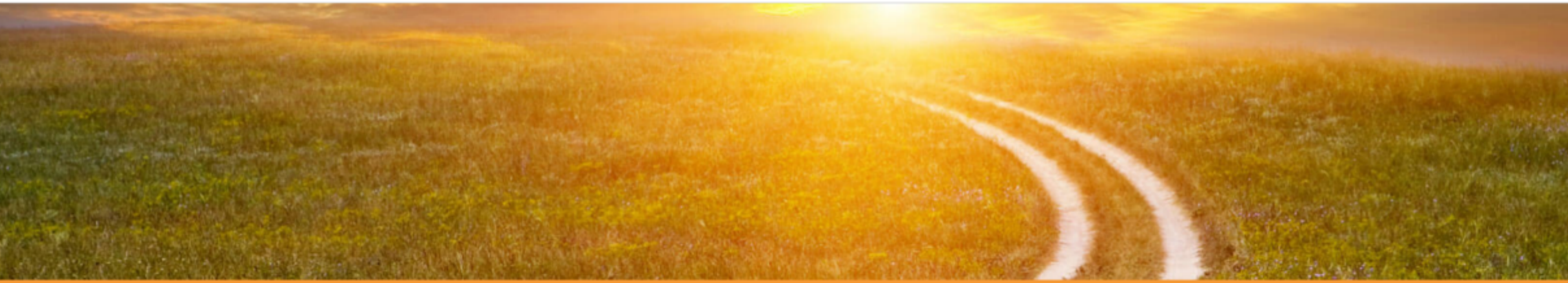

MY REFLECTIONS

Today
Sat | Jul 13

Take 1-2 minutes to reflect on your day. You can choose to focus on one or all sections. If you miss a day, not to worry – you can go back up to 2 days to reflect. You will quickly see how empowering reflecting can be. This is the key to getting started living the life you dream!

WATCH TUTORIAL VIDEO

ALL MY MIND MY NUTRITION MY FITNESS MY BIOMETRICS

Short-Term Goals

- Did my actions today support my short-term goals?
  1. I will stretch every night before bed. ◉ yes ○ no

Long-Term Goals

- Did my actions today support my long-term goals?
  1. I will train for a marathon this year. ◉ yes ○ no

Energy (1=Low 5=High)

- Today my energy level was 5

Focus (1=Low 5=High)

- Today my level of focus was 4

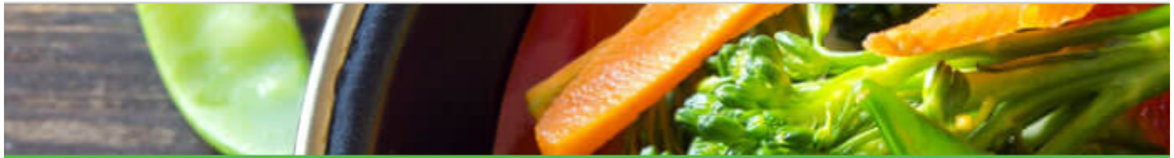

MY RECIPES

Want to take a recipe that helps you live your Viva life to the next level?
Email the recipe (along with a picture of the dish) to support@myvivaplan.com, and we will add our Viva touch!
This includes a re-vamp of the recipe, nutrient analysis and the chance to have it added into our recipe repertoire
– WITH CREDIT GIVEN FOR YOUR INSPIRATION!

Baked Goods | Breakfast | Dips/Sauces | Entrée | Meal Prep | Quick Meals | Salads | Side Dishes | Slow Cooker | Snack | Soups/Stews | Treats/Beverages |

Vegetarian/Vegan

MY FAVOURITES ADD NEW RECIPE Search...

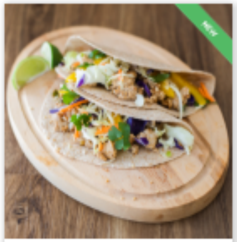

Asian Tacos

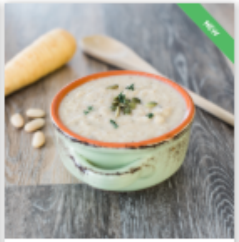

Roasted Parsnip and White Bean Soup

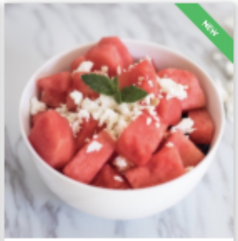

Watermelon Salad

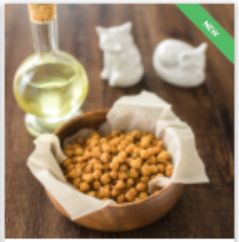

Crispy Chili Chickpeas

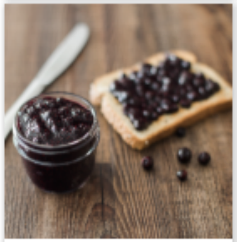

Blueberry Chia Seed Jam

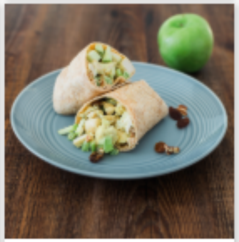

Apple and Turkey Curry Wrap

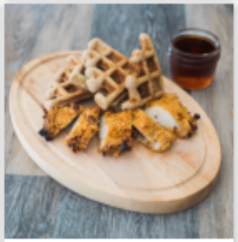

Chicken and Waffles

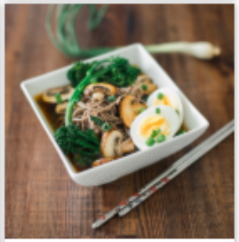

Miso Soup with Buckwheat Soba Noodles

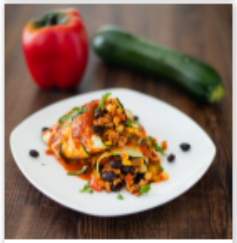

Zucchini and Turkey Enchiladas

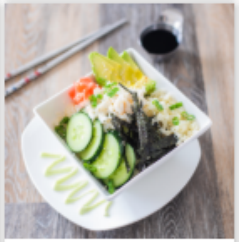

Crab Sushi Bowl, with Cauliflower [illegible]

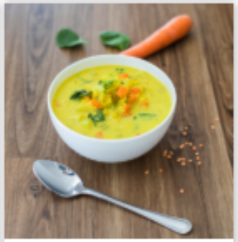

Turmeric Lentil Soup

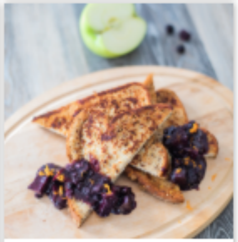

French Toast with Apple Blueberry [illegible]

MY DAY MY MIND MY NUTRITION MY FITNESS COACHING LIBRARY MY ACCOUNT Roshni SIGN OUT UPLOAD

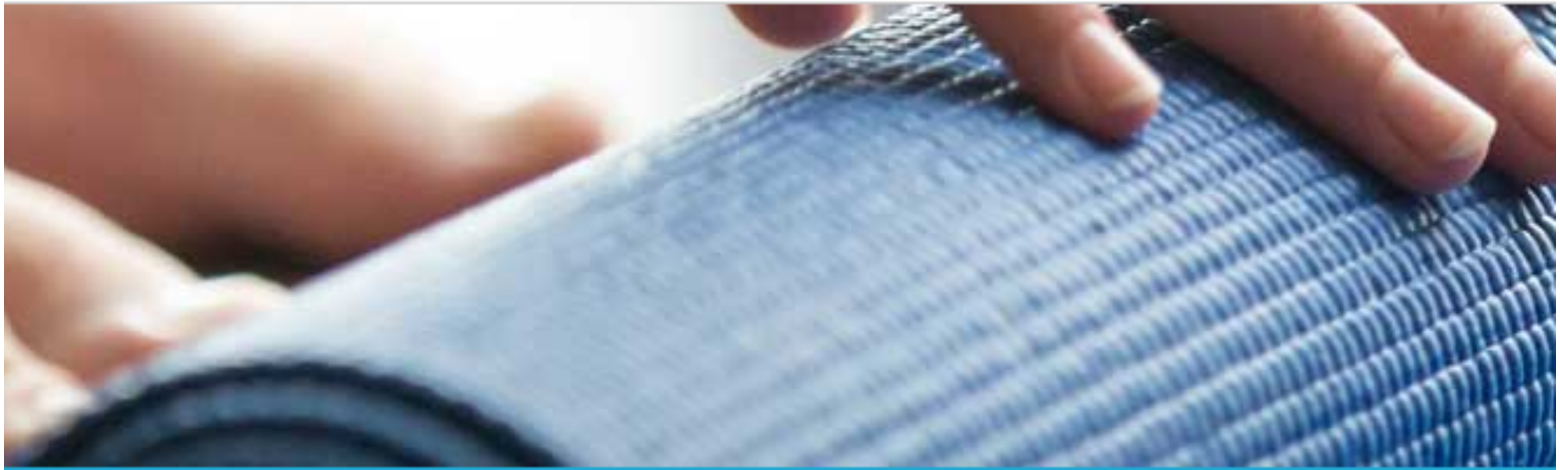

MY VIVA YOGA

Sat | July 13

Create routine Saved routine

Fitness Level * Beginner

Workout Length * 5 minute

Routine * 5 Minute Meditation

*REQUIRED

FITNESS WAIVER:

*By checking this box you are agreeing to the **FITNESS WAIVER** for My Viva Plan.*

☐ I AGREE

START
