# Supplementary figures and images for: The Impact of a Web-Based Mindfulness, Nutrition, and Physical Activity Platform on the Health Status of First-Year University Students: Protocol for a Randomized Controlled Trial
Source: JMIR Res Protoc. 2021 Mar 10;10(3):e24534. doi: 10.2196/24534 (PMC7991982; doi:10.2196/24534)

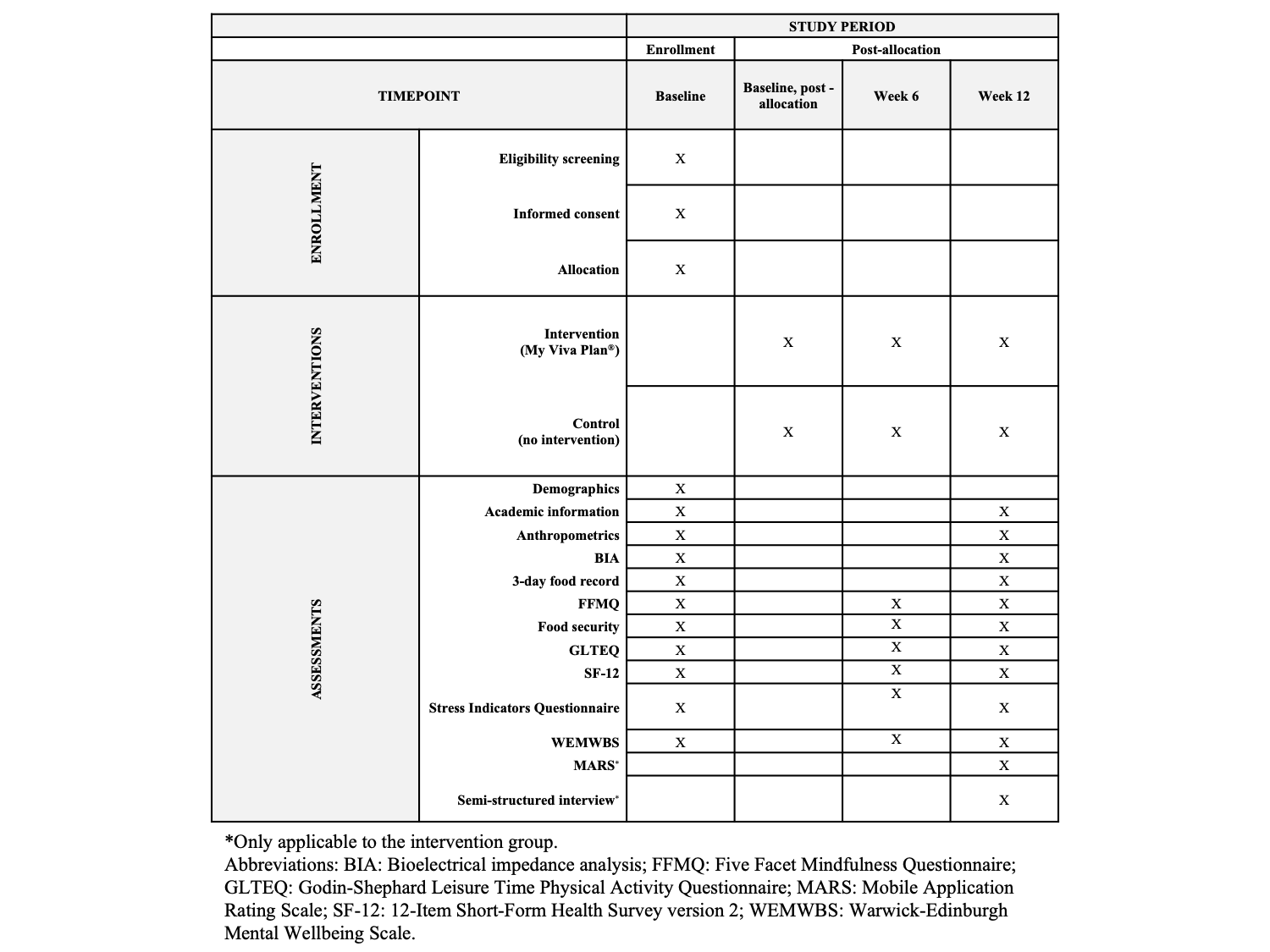

Supplement: Multimedia Appendix 2 [file resprot_v10i3e24534_app2.png]
